# Generation of Genic Diversity among *Streptococcus pneumoniae* Strains via Horizontal Gene Transfer during a Chronic Polyclonal Pediatric Infection

**DOI:** 10.1371/journal.ppat.1001108

**Published:** 2010-09-16

**Authors:** N. Luisa Hiller, Azad Ahmed, Evan Powell, Darren P. Martin, Rory Eutsey, Josh Earl, Benjamin Janto, Robert J. Boissy, Justin Hogg, Karen Barbadora, Rangarajan Sampath, Shaun Lonergan, J. Christopher Post, Fen Z. Hu, Garth D. Ehrlich

**Affiliations:** 1 Allegheny General Hospital, Allegheny-Singer Research Institute, Center for Genomic Sciences, Pittsburgh, Pennsylvania, United States of America; 2 Computational Biology Group, Institute of Infectious Disease and Molecular Medicine, University of Cape Town, Cape Town, South Africa; 3 Department of Internal Medicine, University of Nebraska Medical Center, Omaha, Nebraska, United States of America; 4 Children's Hospital of Pittsburgh, Pittsburgh, Pennsylvania, United States of America; 5 Abbott Molecular IbisBiosciences Division, Carlsbad, California, United States of America; 6 Department of Microbiology and Immunology, Drexel University College of Medicine, Allegheny Campus, Pittsburgh, Pennsylvania, United States of America; 7 Department of Otolaryngology Head and Neck Surgery, Drexel University College of Medicine, Allegheny Campus, Pittsburgh, Pennsylvania, United States of America; New York Medical College, United States of America

## Abstract

Although there is tremendous interest in understanding the evolutionary roles of horizontal gene transfer (HGT) processes that occur during chronic polyclonal infections, to date there have been few studies that directly address this topic. We have characterized multiple HGT events that most likely occurred during polyclonal infection among nasopharyngeal strains of *Streptococcus pneumoniae* recovered from a child suffering from chronic upper respiratory and middle-ear infections. Whole genome sequencing and comparative genomics were performed on six isolates collected during symptomatic episodes over a period of seven months. From these comparisons we determined that five of the isolates were genetically highly similar and likely represented a dominant lineage. We analyzed all genic and allelic differences among all six isolates and found that all differences tended to occur within contiguous genomic blocks, suggestive of strain evolution by homologous recombination. From these analyses we identified three strains (two of which were recovered on two different occasions) that appear to have been derived sequentially, one from the next, each by multiple recombination events. We also identified a fourth strain that contains many of the genomic segments that differentiate the three highly related strains from one another, and have hypothesized that this fourth strain may have served as a donor multiple times in the evolution of the dominant strain line. The variations among the parent, daughter, and grand-daughter recombinant strains collectively cover greater than seven percent of the genome and are grouped into 23 chromosomal clusters. While capturing *in vivo* HGT, these data support the distributed genome hypothesis and suggest that a single competence event in pneumococci can result in the replacement of DNA at multiple non-adjacent loci.

## Author Summary

Bacterial infections have long been studied using Koch's postulates wherein the paradigm is that a single clone leads to a given infection. Over the past decade, it has become clear that chronic bacterial infections often do not fit this paradigm. Instead these are associated with the presence of multiple strains or species (polyclonal) of bacteria that are organized into highly structured communities, termed biofilms, which can persist in the body and are recalcitrant to antibiotic treatment. In addition, there is extensive evidence that bacteria can incorporate genes from neighboring bacteria into their own genomes. This process can produce new strains and is known as horizontal gene transfer. In this study, we investigated for the first time, the tempo and relevance of gene transfer among bacterial strains of *Streptococcus pneumoniae* during a naturally occurring chronic childhood infection. We identified extensive gene transfer among multiple infecting strains, by sequencing of isolates recovered sequentially over a seven-month period. This gene transfer may serve as a counterpoint to the host's adaptive immune response and help explain the phenomenon of bacterial persistence, since, as occurs with some chronic viral and parasitic infections, the immune system may become overwhelmed by a set of related strains.

## Introduction

Horizontal gene transfer (HGT) is a fundamental process in bacterial genome evolution [1]. In the context of infections it can provide pathogenic bacteria with ready access to crucial resistance determinants or virulence factors. Analysis of whole genome sequences (WGS) of multiple isolates from a single bacterial species have revealed extensive population-wide differences among strains in multiple species [2]–[4]. The differences among strains can occur through small-scale mutations affecting only a few base pairs (substitutions, deletions, insertions) or by HGT where DNA segments from hundreds to hundreds of thousands of bases can be incorporated from another organism's genome without the recipient being its offspring. HGT, via transformation, transduction, or conjugation, can lead to the acquisition of entirely new sequences, as well as sequences that are homologous to existing DNA. The transfer of DNA via homologous recombination (HR) leads to the replacement of a region of the genome of a recipient cell by the corresponding region from the donor cell [5]. HR can lead to differentiation by incorporation of identical genes containing single nucleotide polymorphisms (SNPs) and/or the insertion and/or deletion of entire genes and operons. In addition, HR can also increase similarity by incorporation of closely related regions [6]. Whereas only rarely are HGT events detected in *Mycobacterium* species [7], they are frequently observed in naturally transformable species such as *Streptococcus pneumoniae* where multi-locus sequence typing (MLST) and theoretical modeling have indicated that recombination rates are 3 to 10 fold higher than DNA polymerase mutation rates [8], [9]. These highly variable HGT rates imply that the relative contributions of HGT and point mutations to the genomic diversification process varies greatly amongst species. In addition to the variability in HGT rates, there may also be extensive disparity of the sizes of genome regions that are replaced. While it is generally assumed that HR involves mainly small regions, experimental work and *in silico* comparison of multiple WGSs of *S. agalactiae* demonstrated that HR can transfer DNA segments of several hundred kilobases [10]. However, the tempo, pattern and relevance of HGT to bacterial strain evolution within natural chronic infections remains poorly understood, with there being only one published study in *Helicobacter pylori* [11]. In this regard, *S. pneumoniae* is ideal for studying the evolution of bacterial genomes in real time because it forms persistent polyclonal biofilms on the mucosal surfaces of the nasopharynx and the middle-ear. Such environments are highly conducive to HGT [12].

*S. pneumoniae* is a gram-positive bacterium, commonly referred to as pneumococcus, which is causatively associated with severe invasive diseases such as meningitis and bacteremia, as well as with many mucosal diseases including pneumonia, sinusitis, and otitis media (OM) [13]. Worldwide, *S. pneumoniae* is estimated to kill annually one million children under the age of five. In Europe and the USA, *S. pneumoniae* accounts for at least 30% of all cases of community-acquired pneumonia admitted to hospitals, and has a case fatality rate of 10–30% [14]. Despite its pathogenic potential, *S. pneumoniae* is a common natural component of the human nasopharyngeal (NP) commensal flora. In developed countries virtually every child becomes an NP carrier of *S. pneumoniae* during the first year of life with a recent study of European day care centers reporting that over 95% of the children were colonized by *S. pneumoniae* at least once during the study with many children showing evidence of polyclonal infection [15], [16].

There are 91 *S. pneumoniae* serotypes and very significant differences with regard to genic (gene possession) diversity and disease-inducing phenotypes both within and among serotypes [4], [17]–[19]. Collectively WGS analyses have support the distributed genome hypothesis (DGH) that posits that there are many genic differences that exist among the individual strains that make up a bacterial species (or infecting population). Thus, there exists a species-level supragenome (pangenome) that is much larger than the genome of any given strain [2], [3] [20], [21]. Previous studies have provided evidence in support of the DGH by demonstrating that fewer than 50% of the total number of *S. pneumoniae* genes that have been identified are found in any individual strain [4]. Both the intense intra-species competition within *S. pneumoniae* biofilms [22]–[25] and the natural capacity of *S. pneumoniae* to undergo transformation by the active uptake of environmental DNA embedded within the extracellular polymeric matrix of these biofilms [26], have likely driven much of the genic diversification of this species. The DGH postulates that the same mechanisms which promote genomic plasticity at the species level also result in the *in situ* creation of clouds of related *S. pneumoniae* strains within chronically infected individuals and that these may act as a potent counterpoint to the host's adaptive immune response [27].

## Results

### Selection and sequencing of *S. pneumoniae* strains from a pediatric patient

As part of an influenza vaccine trial, NP sampling was performed on pediatric patients presenting with flu-like respiratory symptoms. Bacteria were recovered, isolated, typed, and frozen from these samples [28]. An 8 month-old child, patient 19, enrolled in this study had 12 clinic visits due to rhinorrhea and/or ear infections over a 7-month period. These included a visit at enrollment and seven subsequent visits during which nasopharyngeal swabs were obtained for bacterial culture (Table 1). All of the bacterial strains recovered from this patient were typed as *S. pneumoniae* and could be divided into one of two MLST types: ST13 or ST2011. As with other bacterial species, MLST-based analyses of *S. pneumoniae* strains enables accurate identification using the allelic profiles of seven housekeeping genes that are strongly correlated with, and indicative of, genome-wide degrees of strain variability (ST13 and ST2011 differ in the sequence of two of these genes, specifically xpt and ddl) [29]. The two distinct MLSTs identified within patient 19 suggest that the child was infected with at least two divergent *S. pneumoniae* strains. Interestingly, half of the ST13 strains were identified as being of serotype 14, and the others were non-typeable (Table 1). The patient 19 isolates are named by their MLST type (ST13 or ST2011), followed by the visit number when they were isolated (v1 through v13).

**Table 1:** Patient's clinical history and clinical, sequencing and assembly information on the *S. pneumoniae* strains.

**Visit No**	1	3	4	5	6	10	12	13
**Date**	5-Oct-99	8-Nov-99	22-Nov-99	9-Dec-99	4-Jan-00	23-Mar-00	19-Apr-00	12-May-00
**Serotype**	14	14	NT	NT	NT	14	NT	NT
**MLST**	13	13*	2011	2011*	13	13	13	13
**Sympthoms**	Enrollment	Mild rhinorrhea Diagnosis: No AOM	Pulling ears, conjunctivitis Diagnosis: Bilateral AOM	Pulling ears, fussy Diagnosis: Right AOM	Pulling ears, decreased appetite, cough, irritable, thick copious rhinorrhea Diagnosis: Bilateral AOM	URI, pulling ears, irritable Diagnosis: Bilateral AOM	Fever, URI Diagnosis: Left AOM, Right OME	Vomiting and Diarrhea x 5 days, not sleeping well, decreased activity Diagnosis: Bilateral AOM
**Strain Name**	ST13v1	ST13v3	ST2011v4	ST2011v5	ST13v6	ST13v10	ST13v12	ST13v13
**Sequence Status**	yes	no	yes	no	yes	yes	yes	yes
**Genbank ID**	ABWQ	n/a	ADHN	n/a	ABWB	ABWA	ABWU	ABWC
**CG%**	39.53	n/a	39.6	n/a	39.6	39.54	39.63	39.65
**Genome Sequence Length (bp)**	2,100,368	n/a	2,086,050	n/a	2,053,197	2,063,728	2,065,452	2,070,802
**Read Coverage**	21	n/a	28	n/a	27	29	23	28
**Sequencing Platform**	FLX	n/a	Titanium	n/a	FLX	FLX	FLX	FLX
**Average Read Length**	252	n/a	358	n/a	217	226	253	261
**No reads assembled**	177304	n/a	167824	n/a	228980	243895	191642	221409
**Newbler Contigs**	87	n/a	113	n/a	97	109	87	81
**Final Contigs after PCR gap closure**	25	n/a	113	n/a	97	109	30	22

*the MLST profiles for ST13v3 and ST2011v5 were determined using the Ibis T-5000 technology.

ST13 and ST2011 differ in the sequence of their ddl and xpt alleles (ST is based on allelic differences of 7 housekeeping genes).

AOM: acute otitis media; OME: otitis media with effusion; URI: respiratory tract infection; n/a: not available.

454 Lifesciences-based pyrosequencing (without paired end analysis) was used to sequence six of these isolates (Table 1). A PCR-based analysis of both of the non-sequenced isolates indicates that they are clones of sequenced isolates as their gene possession profiles are identical (data not shown). The complete genomic sequences of the six sequenced isolates have been deposited in GenBank and are also available at the Strepneumo database http://strepneumo-sybil.igs.umaryland.edu/. The genomes have an average size of 2,070±17 Kb and a GC content of 39% (Table 1). The Microbial Genome Annotation Tools and Genome Annotation Pipeline from NCBI were used to predict and annotate the coding sequences (CDSs) (http://www.ncbi.nlm.nih.gov/genomes/static/Pipeline.html). The average number of CDSs per strain is 2250 (Table 2).

**Table 2:** Summary of CDSs and their organization into orthologous clusters.

Strain	CDSs	Orthologous Clusters	Distributed Clusters	Core Clusters
BS292	2290	2158	77	2077
BS457	2225	2141	64	2077
BS458	2254	N/A	N/A	N/A
BS293	2242	2125	44	2077
BS397	2242	N/A	N/A	N/A
BS455	2182	2196	116	2077
All	13435	2250*	173*	2077

*after curation using PCR; N/A: non applicable.

### Evidence of recombination among strains from WGS comparison

Global comparisons of the WGS of these six isolates revealed that two pairs were essentially identical (i.e. there are four strains with two isolates each of two of the strains) despite being sampled 23 days apart (ST13v12 and ST13v13) and ∼5 months apart (ST13v1 and ST13v10) (Text S1 and Text S2, respectively). Thus, it can be inferred that the first ST13 strain that was isolated persisted for at least 5 months without any detectable evidence that it was an HGT recipient.

**Figure 1:**
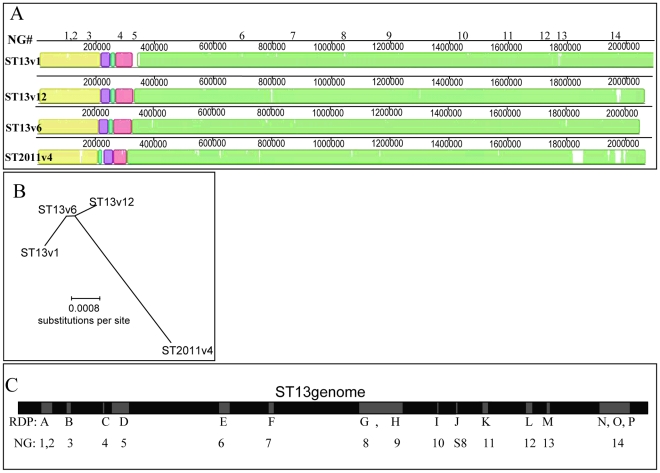
Demonstration of recombination events among *Streptococcus pneumoniae* genomes isolated from a single patient. (A) MAUVE alignment of the WGSs of four *S. pneumoniae* isolates showing four distinct strains. Colored blocks highlight regions that are homologous and free from genomic rearrangement. White areas illustrate degrees of average conservation that exist within corresponding genome regions (more white  =  less conservation). Neighbor group (NG) numbers above the schematic denote the positions of clustered SNP groupings. (B) Maximum likelihood phylogenetic tree (given the best fit model  =  F84+G_4_) expressing the relationship between the four genomes and demonstrating that ST2011v4 is the most divergent of these strains and that differences exist among the ST13 strains. (C) Schematic showing the relative position of recombination events (gray boxes) within the ST13 genome (black), as predicted by RDP3 and NG analyses.

The WGS of the four genically distinct strains (ST13v1, ST2011v4, ST13v6, and ST13v12) were aligned using the progressive Mauve feature in the MAUVE genome alignment software [30]. To visualize the genomic differences, a similarity plot was generated from this alignment (Fig. 1A, white areas represent areas of low conservation). To assess the phylogenetic relationships among these strains, a maximum likelihood tree was created from the alignment using PHYML [31] as implemented in Recombination Detection Program (RDP) [32] (Fig. 1B). Both figures show that strain ST2011v4 is the most distant among the strains isolated from this patient, and that smaller differences also exist among the ST13 strains.

To investigate the role of HGT in differentiation of these strains, we created a specially modified version of RDP (RDP3) capable of comparing full-length bacterial genomic sequences and identifying recombination sequences and their breakpoints (revision 42–2; freely available from http://darwin.uvigo.es/rdp/rdp.html). RDP3 implements a variety of published recombination detection methods to determine statistical evidence of recombination [32]. To avoid any sequencing artifacts, all base pairs with a sequencing quality score of less than 40 (a probability of >1∶10^4^ that they were incorrectly called) were eliminated from this and all subsequent analyses. The final RDP analysis identified evidence of 16 statistically significant recombination events among the four strains (Fig 1C, Table 3; with all detected events identified unambiguously by at least five out of seven independent recombination signal detection methods). This analysis suggests that 459 Kb of genomic sequence was exchanged among the analyzed strains by recombination. These segments vary in size from 0.4 kb to 235 Kb, with a mean size of 28 Kb and a median size of 13 Kb.

**Table 3:** Recombination fragments predicted by RDP3 analysis, their corresponding probabilities after Bonferroni correction, and estimated size in base pairs.

RDP event	RDP Probability (after Bonferroni correction)	Size of HGT on ST13v1 based on RDP analysis
A	1.16 E-76	36868
B	2.30E-98	12845
C	9.28E-47	3506
D	2.31E-70	15544
E	3.87E-61	6235
F	2.84E-59	14372
G	4.29E-121	34671
H	4.30E-17	235426
I	3.66E-105	6314
J	1.83E-36	402
K	2.50E-82	16599
L	9.45E-295	14674
M	9.31E-11	7583
N	1.69E-123	4715
O	1.03E-11	2044
P	1.17E-80	47900

Comprehensive SNP analyses were used to further investigate the differences amongst these four strains. MAUVE was used to generate a list of all 11470 SNPs (Table S1). The majority of the SNPs (71%) are identical among the three ST13 strains but variable in relation to the ST2011v4 strain (rows 3310–11474 in Table S1). Nonetheless, 28% (3306 of 11470) of the SNPs show differences among one or more of the ST13 strain pairs (first 3308 rows in Table S1). To determine the relative positions of these 3306 ST13 SNPs, they were sorted based on their chromosomal placement. The sorted list was manually curated to group together SNPs that a) share the same distribution across strains (that is, the same strain contains the variable nucleotide) and b) are located within an area where there is high concentration of SNPs (from 4 to 68 SNPs/Kb – as opposed to isolated SNP found at levels <0.2SNPs/Kb); such groups are hereafter referred to as neighbor groups {NG} (Fig. 1C). The NG breakpoints were selected as areas that demark the transition between highly conserved regions (less then 0.2 SNPs/Kb) and divergent regions (more then 4 SNPs/Kb). Ninety five percent of these 3306 ST13 SNPs were organized into 23 distinct chromosomal NGs. Fourteen NGs are larger then 500 bp and nine are smaller then 500 bp (Tables 4 and 5 respectively, a detailed list of all the SNPs and their organization into NGs is illustrated in the first 3308 rows of Table S1). NG analysis suggests that HGT has led to the exchange of at least 156 Kb between strains ST13v1 and ST13v12.

**Table 4:** Genic and allelic differences identified with large (>500 bp) regions of horizontal gene transfer among *S. pneumoniae* isolates from a single patient.

NG#	Corresponding RDP	Start Position on ST13v1	End Position on ST13v1	No SNPs on WGS	No SNPs on CDSs	No Distributed Genes	Size of HGT on ST13v1 based on SNP analysis	Likely Recipient:Donor Strains	Likely Recombinant
1	part of A	78193	78961	58	58	0	768	ST13v6 : ST2011v4	ST13v12
2	part of A	102716	107068	138	70	0	4352	ST13v1: ST2011v4	ST13v6
3	B	165124	169392	125	124	0	4268	ST13v6 : ST2011v4	ST13v12
4	C	294136	295217	55	55	0	1081	ST13v6 : ST2011v4	ST13v12
5	D	321068	350885	630	525	17	29817	ST13v1: ST2011v4	ST13v6
6	E	689372	699927	54	33	0	10555	ST13v6-like: ST2011v4	ST13v6
7	F	871647	876989	30	24	0	5342	ST13v6 : ST2011v4	ST13v12
8	part of G	1207132	1226947	159	102	0	19815	ST13v6 : ST2011v4	ST13v12
9	part of H, part of G	1311469	1313700	16	6	0	956	ST13v6 : unknown	ST13v12
10	I	1460234	1460971	58	0	0	737	ST13v1-like: ST2011v4	ST13v1
11	K	1621147	1627678	51	49	0	6531	ST13v6 : ST2011v4	ST13v12
12	L	1767032	1779723	311	148	5	12691	ST13v1: ST2011v4	ST13v6
13	M	1841607	1844874	84	1	0	3267	ST13v6-like: unknown	ST13v6
14	N,O,P	1978118	2034639	1274	1020	24	56521	ST13v6 : unknown	ST13v12
Total				3043	2215	46	156701		

NG  =  neighbor groups, i.e contiguous genes that moved *en bloc* as part of a single horizontal gene transfer event. RDP  =  recombination blocks predicted by RDP3. Regions 1, 3, 4, 7, 8 and 11 likely were transferred during a single competence event; similarly for the regions 2, 5, and 12; both by exchanging DNA with strain ST2011v4 (or ST2011v4-like).

**Table 5:** Genic and allelic differences identified within small (<500 bp) regions of horizontal gene transfer among *S. pneumoniae* isolates from a single patient.

sNG	Start Position on ST13v1	No SNPs on WGS	Size of HGT on ST13v1 based on SNP analysis	Identical Pair of ST13 strains	Strain resembling ST2011v4
1	5275	12	21	unknown (ST13v12 missing sequence)	ST13v6
2	373782	6	15	unknown (ST13v6 missing sequence)	ST13v1
3	415728	4	5	ST13v1 = ST13v12	ST13v1, ST13v12
4	469722	55	134	ST13v6 = ST13v12	ST13v1
5	769198	10	449	unknown (ST13v6 missing sequence)	ST13v1
6	814505	74	317	ST13v1 = ST13v12	none
7	1102745	28	355	unknown (ST13v6 missing sequence)	none
8	1520772	18	351	ST13v6 = ST13v1	ST13v12
9	1603848	9	55	ST13v1 = ST13v12	ST13v6
Total		216	1701		

The analyses with RDP3 (statistical tool package) and NG (manually curated grouping of SNPs based on pattern and localization) predict very similar recombination events (Fig 1C, Table 4). The major differences identified by these two methods are in the position of the recombination breakpoints, and thus the size of each event. The NG method is extensively curated and overall leads to the most conservative estimate (see methods and Text S3). The MAXCHI method used by RDP to infer breakpoint positions identifies breakpoints as the midpoint between the two phylogenetically informative SNPs bounding the borders of identified recombinant regions. As a result the RDP estimated bounds of the recombinant regions are less conservative than the manually curated estimates because they include numerous sites on the 5′ and 3′ ends of the regions that are identical between the identified parental sequences. Regardless of the method selected for analysis, the WGS comparisons suggests that: (1) four distinct strains were isolated from one patient; (2) these strains fall into two groups, three ST13 isolates and one ST2011 isolate; (3) the differences among the ST13 strains are grouped into multiple chromosomal regions, (4) these regions are the result of multiple recombination events.

### Genic differences within the recombinant segments

The RDP3 and NG WGS comparison methods are alignment based, and do not focus on DNA regions that include genic differences (presence/absence of CDSs) since these do not align to other sequences. To analyze these differences, we compared all predicted coding sequences (CDSs) from the six genomes. These CDSs were organized into 2250 orthologous gene clusters as described in [4] and further divided into 2077 core and 173 distributed gene clusters (Table 2). A distributed cluster was defined as any orthologous gene cluster not present in all strains, and as such represents one of the genic differences among strains (complete list in Table S2). There are a total of 126 distributed gene cluster differences between ST2011v4 and the other three strains. These include 37 genes present only in the ST13 strains, and 89 genes present only in the ST2011v4 strain (Table S2). Among the three ST13 isolates there are only 47 genic differences in total. ST13v1 differs from the other strains by 23 genes (18 genes present and 5 genes missing), while ST13v12 differs by 24 genes (2 genes present and 22 genes missing) (Table S2). There were no genic differences between ST13v1 versus ST13v10 or between ST13v12 versus ST13v13. This is consistent with the WGS comparisons that identified these isolate pairs as being nearly identical, thus corroborating the hypothesis that these clones have persisted in the patient without *detectable* HGT. A previous study showed that the number of genic difference between pairs of independently isolated *S. pneumoniae* genomes ranged from 160 to 629 [4], suggesting that all strains isolated from this patient are more closely related than most independent isolate pairs. For comparison, the difference between a clinical strain (D39) and its lab derivative (R6) was 35 genes [4], similar to the number of differences between the ST13 strain pairs. Forty six of the 47 ST13 distributed genes are grouped into three of the recombination regions; 17 belong to the type 14 capsule locus within NG5 (RDP D), 5 belong to NG12 (RDP L), and 24 belong to NG14 (RDP P) (Table 4, and Table S3 provide the annotations for these genes and their relative positions within the recombinant regions). The high degree of genic similarity shared by the CDSs within the ST13 strains, and the positioning of the genic differences within the predicted recombination regions complements the results from the WGS comparisons, suggesting that these strains diverged from each other by multiple recombination events.

### The relative distance amongst the ST13 strains suggests they are not independent isolates

**Figure 2:**
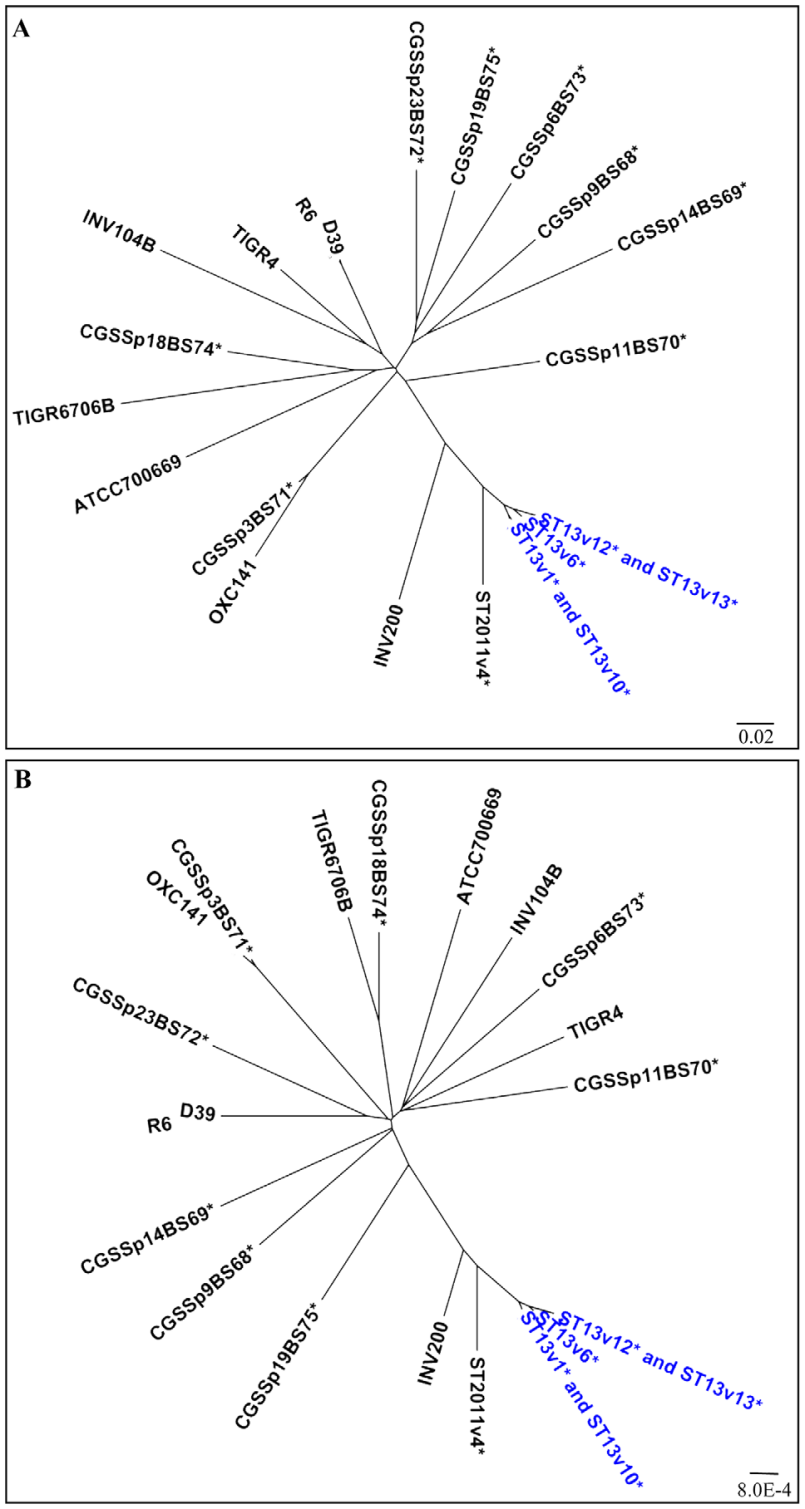
High degree of genomic similarity among ST13 strains. Grouping of 22 *S. pneumoniae* isolates based on (A) numbers of distributed genes and (B) numbers of variable core alleles. These graphs provide a measure of the genic or alleic distance among isolates without making any inference regarding their phylogeny, since the high rates of recombination within the population can interfere with population-wide phylogenetic results. The high degree of similarity among the ST13 strains isolated from patient 19 suggests that these strains have evolved within this patient and are not the result of independent infections. Further details on the strains are described in Hiller *et al* [4].

For a population-wide perspective, we quantified the allelic and genic differences of 22 *S. pneumoniae* strains, including the 6 strains in this study. The WGS of the remaining 16 has been previously published [4]. These comparisons group strains based on either their genic or allelic content, but do not account for their phylogenetic relationships since high recombination rates can abrogate genome-wide phylogenetic signals [33]. The genic distance measured between genomes was defined as the number of distributed gene clusters shared (both strains contain the gene) or not shared (neither strain contains the gene) by a given strain pair, divided by the total number of distributed gene clusters (Fig. 2A)[34]. The allelic distance measure was based on the variation among the core gene clusters [34] (Fig. 2B). These graphs show that the ST13 strains are more closely related then most other isolates collected from independent infections. Note again that R6 is a lab derivative of D39 and therefore these strains are not independent isolates. The only other similarly closely related strains is a pair of serotype 3 ST180 strains (OXC141 and CGSSp3BS71) that show allelic distances comparable to that of the ST13 strains. Importantly, other strains from the same study, all collected in the same hospital in Pittsburgh over the same time period are highly genetically variable (asterisks in Figs. 2A and 2B), demonstrating that the similarity among the ST13 strains isolated from patient 19 is unlikely to be an effect of the isolation locale. These data strongly suggest that the ST13 strains are more similar than would be expected had they been isolated from independent infections. In contrast, the larger distance between ST2011v4 and the ST13 strains suggests that ST2011v4, or a highly related strain, was acquired during an independent infection.

### Identification of ST2011v4 as a DNA donor for ST13 strains

**Figure 3:**
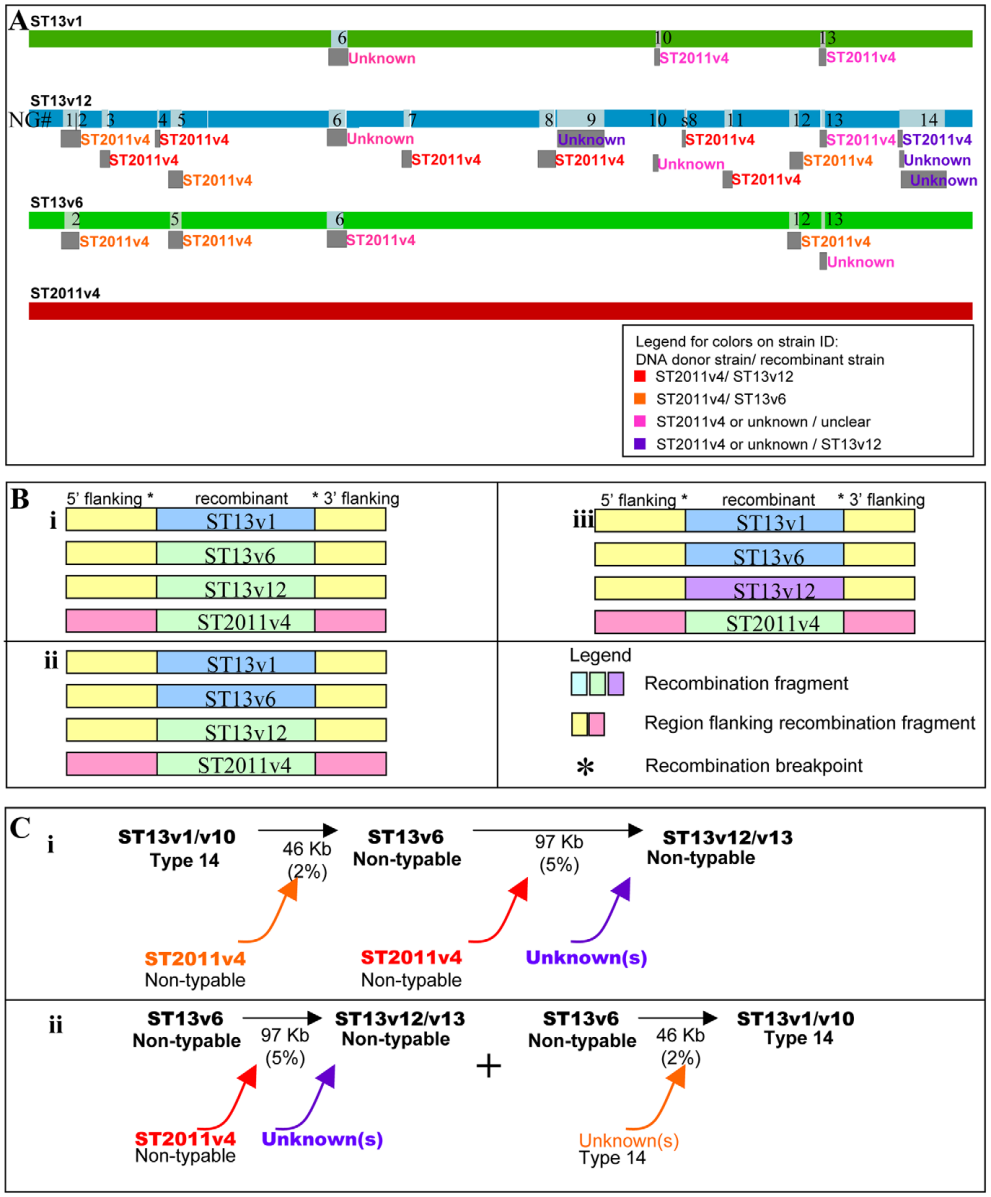
Identification of DNA donors for recombinant ST13 isolates. (A) RDP3-generated schematic of four genomes indicating evidence of recombination with neighbor group (NG) results superimposed. Light gray boxes within the whole genome sequence (WGS) schematic represent recombination events, and corresponding NG numbers have been inserted. Dark gray boxes are positioned below likely recombinant strains and are labeled with the most likely DNA donor for each transferred region. (B) Schematic illustrating the various patterns found in both the recombinant regions and surrounding areas. (i) Recombination fragments that are identical among ST2011v4, ST13v6, and ST13v12 while the surrounding regions are identical only among the ST13 strains (but contain SNPs relative to ST2011v4) (representative of NGs 5 and12 and corresponding to RDP D and L); (ii) Recombination fragments that are identical only between ST2011v4 and ST13v12 while the surrounding regions are identical only among the ST13 strains (representative of NGs 1,3,4,7, and 11 and corresponding to RDP part of A, B,C, F and I); (iii) Recombination fragments that differ between ST13v12 and all other strains (representative of NGs 9 and 14 and corresponding to RDP part of G and N, O and P). (C) Schematic outlining possible recombination events that may have led to the creation of the strains isolated from the patient. (i) most and (ii) next-most parsimonious. Black arrows move from the major parental strain to the recombinant, colors highlight likely DNA donor strains. The conservative estimate for the size of the recombination fragments is marked below the arrows with the corresponding percentage of the WGS in parenthesis.

The most likely donor and recombinant strains were selected based on RDP3 predictions, as well as SNP patterns from all four unique patient 19 strains where the recombinant regions between the donor and recombinant strains must be virtually identical. Fig. 3A shows the diagram generated using RDP3, with NG results superimposed as numbers within the light gray boxes. Dark gray boxes under the WGS schematic are labeled with the name of the most likely donor strain and located under the schematic of the most likely recombinant strain. In a few cases where multiple options for the recombinant strain are probable, they are all represented. Results suggest that for at least 9 recombination events (red and orange in Fig. 3A) ST2011v4 is the most likely DNA donor, and ST13v6 and/or ST13v12 are the most likely recombinants.

ST2011v4 is identical in many of the recombination segments to one or more ST13 strains (green in Fig. 3Bi, ii). This suggests that either a) ST2011v4 served as DNA donor for these recombination segments, or b) an un-sampled strain served as a DNA donor leading to modification in one or more of the ST13 strains (blue Fig. 3Bi,ii) in a region where ST2011v4 is identical to a subset of the ST13 isolates (green Fig 3Bi,ii). Importantly, the regions surrounding most recombination segments are virtually identical among all three ST13 strains but variable (containing many SNPs) relative to ST2011v4 (yellow versus pink in Fig. 3Bi, ii). Table S3 displays the allelic and genic differences within the recombination fragments, as well as their surrounding areas (labeled W or S, respectively), and Table S1 shows the SNPs surrounding the recombinant region highlighted in yellow (within rows 3310–11474). Two observations provide compelling evidence for the first option where ST2011v4 acted as a DNA donor to ST13 strains. The first observation is the *regional* genomic similarity between ST2011v4 and subsets of the ST13 strains in the recombinant region (green Fig. 3Bi,ii). The second observation is the genomic identity among the ST13 strains but *not* ST2011v14 on the regions surrounding the recombinant fragments (yellow versus pink in Fig.3 Bi,ii). Moreover, the synteny among all four strains in and around the recombination breakpoints suggests that HR is the most likely operative mechanism.

The scenario involving the least number of strains and recombination events that explain the genomic sequences isolated from this patient is illustrated in Fig. 3Ci. Here, ST13v6 evolved from ST13v1 through the acquisition of NGs 2,5, and 12 from ST2011v4 (ST13v6 and ST2011v4 are identical in these regions yet differ from ST13v1 - Fig. 3Bi). Using the most conservative recombination estimates, these three regions add up to 46.8 Kb, include 22 distributed genes, and differ by 1079 SNPs (strain name in orange in Fig. 3A and sizes in Table 4). Subsequently, ST13v12 evolved from ST13v6 through the acquisition of NGs 1,3,4,7, 8, and 11 from ST2011v4 (ST13v12 and ST2011v4 are nearly identical in these regions yet differ from ST13v1 and ST13v6 - Fig. 3Bii). Using the most conservative recombination estimates, these six HGT regions sum to 37.8 Kb, and differ by 478 SNPs (strain name in red Fig. 3A and sizes in Table 4).

While the scenario illustrated in Fig. 3Ci is the most likely explanation for the evolution of the sequenced strains, we are unable to exclude the possibility that a different, albeit less parsimonious, pattern of HGT might have yielded the observed genetic variation (Fig. 3Cii). In this second scenario, ST13v1 may be a recombinant having arisen from transfer of DNA (NGs 2, 5, and 12) from an unknown parental donor into either ST13v6, or a highly related strain.

Collectively, these data place ST13v6 as a genomic intermediate between ST13v1 and ST13v12, since it shares 3 recombination events in common with ST13v12 (NGs 2, 5, and 12) but lacks evidence of additional events (NGs 1,3,4,7, 8, and 11). Notably, this model of recombination events is consistent with the time of isolation of these strains.

### Evidence for additional DNA donor(s) and mutation events

**Figure 4:**
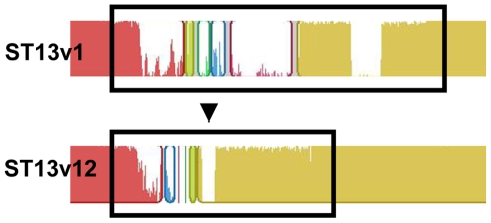
Illustration of a region transferred by HGT. MAUVE alignment of the genome region of ST13v1 and ST13v12 corresponding to NG14 (containing RDP N, O and P). Colored blocks represent NG14 regions that are homologous; a similarity plot inside each block portrays the average degree of conservation in that region. Black boxes bound sites displaying the most obvious differences between the 57 Kb NG14 region of ST13v1 and the 34 Kb NG14 region of ST13v12.

Not all of the recombination fragments can be explained by the genetic exchanges occurring among the four unique sampled strains. There are 2 recombinant regions (NGs 9 and 14- RDP H, O and P) that must have been acquired by ST13v12 from an unsampled donor, as these regions are unique with respect to all of the sequenced strains including ST2011v4 (purple in Fig. 3A and 3Biii). NG 14 (RDP O and P) is ∼50 Kb, differs from the other ST13 strains by 24 distributed genes and ∼1200 SNPs (Fig. 4). Importantly, this region is also unique with respect to the un-sequenced ST13v3 and ST2011v5 strains as shown by PCR-based sequencing of the target regions, demonstrating that these strains cannot have served as donors. NG9 is also unique with respect to all of the other sequenced and unsequenced strains isolated from this patient suggesting, as for NG14, an origin from an unsampled strain.

Further differences among the strains suggest that additional HGT events affecting a smaller number of loci have also occurred during this infection. The 5′ four Kb in NG14 (RDP N) varies between ST13v1 and ST13v6, and the 3′ end of NG8 varies between ST2011v4 and ST13v12 suggesting the occurrence of additional DNA exchange events at these regions. Also, there are an additional three regions where ST2011v4 may have served as a donor (pink in Fig 3A). In NG10 (RDP I), ST13v1 resembles ST2011v4 but not ST13v12 (data for ST13v6 is missing in this region). In NG13 (RDP M), ST13v6 differs from all strains while ST13v1 and ST13v12 resemble ST2011v4 suggesting that ST13v6 underwent an additional HGT event in this region with another donor, and therefore the direct antecedent of ST13v12 incurred an additional change creating the ST13v6 genome. Finally, for the majority of the SNPs in NG6 (RDP E), ST13v6 differs from the other ST13 strains but resembles ST2011v4 suggesting yet another HGT event from a relative of ST2011v4.

In addition to the large (>500 bp) recombinant regions, we identified an additional 9 regions that are <500 bp (Table 5). These nine small NGs (sNG) have a variety of SNP patterns (detailed SNPs in Table S1). Finally, minor differences among the strains also implicate other mutation mechanisms. Differences in the number of repeats in the glucan binding domain of pneumococcal protein A genes is suggestive of DNA polymerase slippage, and differences in restriction endonucleases resemble DNA inversions between S subunits (Text. S1b and Fig. S1, respectively) [35].

### Phenotypic differences amongst the ST13 strains

**Figure 5:**
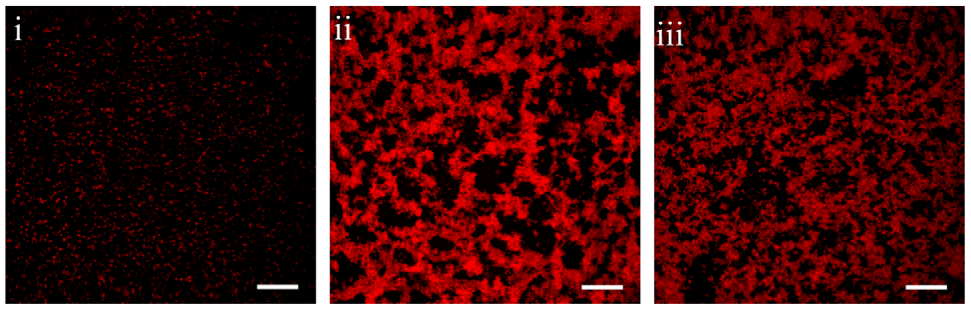
Phenotypic differences among ST13 strains. Biofilm development after 5 days on polystyrene plates by clinical isolates (i) ST13v1, (ii) ST13v6, and (iii) ST13v12. Images are maximum projections of reconstructed confocal stacks consisting of a series of x–y sections. Cells were stained with Syto59 [26]. Scale bar  = 30 µm.

Given the polyclonal nature of infection it is not possible to correlate with a high degree of confidence the patient's symptoms observed during any particular visit to the bacterial strain isolated from samples collected at that visit. Nonetheless, it is noteworthy that the appearance of the nontypeable (NT) strain ST2011v4 correlates with the beginning of a severe bout of acute otitis media (diagnosis listed in Table 1). It is therefore conceivable that either this strain itself or sequences horizontally transferred from this strain into the ST13 strain may have had an influence on virulence. To investigate how the genetic differences amongst these strains may have affected their biology we compared the capacities of the ST13 strains to form biofilms. Biofilm-formation is thought to be important for persistence following nasopharyngeal colonization and for the establishment and maintenance of chronic mucosal infections such as otitis media with effusion [36], [37]. Confocal images of biofilms produced *in vitro* by the ST13 strains after 1,3, and 5 days of growth show that the two unencapsulated strains ST13v6 and ST13v12 make much more robust biofilms when compared to ST13v1, the capsular type 14 strain (Fig. 5).

### Annotation of neighboring group regions

The differences between the ST13 strains are contained within 150 genes: 46 distributed genes and 104 core genes with allelic differences that collectively contain ∼2200 SNPs (labeled “W” in Table S3). The CDS that differentiate ST13v1 from both ST13v6 and ST13v12 are located within NGs 2, 5 and 12 (RDP part of A, D, L) (Table 4). NG5 (RDP D) on ST13v1 encoded the type 14 capsular genes as well as adjacent allelic core genes (Table S3). In the corresponding region, the non-typeable ST13v6, ST13v12 and ST2011v4 have lost the capsular genes yet carry two genes that are not found in ST13v1. Within NG2 (part RDP A) modifications to pneumococcal surface protein A (*PspA*), a virulence gene that encodes a choline-binding protein associated with resistance to fixation of complement [38] and with binding human lactoferrin [39], also differentiate ST13v1 from ST13v6/ST13v12. Of note, within ST13v12 and ST13v13 there were various nucleotide polymorphisms within *PspA* that were not obviously derived through HGT suggesting that this locus is under strong selective pressure (Text S1 and Table S3). Within NG12 (RDP L) there are three distributed genes in ST13v1 surrounded by 16 allelic core genes, which can be organized into four operons, two of which include only hypothetical proteins. The observed HGT event within this region resulted in ST13v6 and ST13v12 having two genes in this region that is absent in ST13v1. One of these genes has been annotated as a possible cell surface protein. A species-wide comparison demonstrated that neither of these genes is shared with any of the other 16 sequenced *S. pneumoniae* strains analyzed in Fig.2.

The NG14 (RDP O) region of ST13v12 is ∼23 Kb smaller than the corresponding regions of ST13v1 and ST13v6 (Fig. 4). This region in these latter two strains contains 44 genes distributed over at least 4 separate operons. The annotations of these genes suggest that they function in the metabolism and/or transport of amino acids, sugars, zinc and glycerol (Table S3). The corresponding smaller region in ST13v12 is missing 22 of these genes, carries two other genes (a beta-galactosidase and a hypothetical protein), and differs from the other ST13 strains by over 1000 SNPs in their shared genes.

## Discussion

*S. pneumoniae* has long been recognized as a major human pathogen with Sir William Osler at the turn of the 20^th^ century referring to pneumococcal pneumonia as “the captain of the men of death”. Shortly thereafter *S. pneumoniae* was shown to be transformable [40] and this observation led directly to the identification of DNA as the hereditary molecule [41]. However, it has only been in the last decade that HGT has been recognized as a significant virulence trait [27]. In this study we analyzed the WGS of six *S. pneumoniae* isolates obtained over ∼7-months from a single pediatric patient presenting with nasopharyngeal and middle-ear disease symptoms. We recovered two pairs of virtually identical genomes over the 13 visits (at visits 1 and 10 and visits 12 and 13) strongly suggesting that some strains persisted within the patient during asymptomatic periods. Moreover, the recovery of other divergent strains at visits 4 and 6 suggests that the clones isolated on visits 1 and 10 were present simultaneously with these divergent strains. Additionally, since recombination between a donor and recipient strain is much more likely if both parental strains are present simultaneously, it can be inferred that this was a polyclonal infection. This is not surprising given ample evidence for polyclonal carriage of *S. pneumoniae* [15], [16]. Thus, while the available experimental samples (single strain isolations at each of 8 visits) did not allow us to fully survey the polyclonal nature of this *S. pneumoniae* infection, or extract the strains present during non-symptomatic periods, the complete genomes of the strains that we did isolate provide evidence of polyclonality and strain persistence in this patient. Collectively, these strains provide an excellent study set for characterizing *in vivo* HGT during polyclonal nasopharyngeal *S. pneumoniae* infection.

The very high degree of similarity amongst the three ST13 strains relative to all other *S. pneumoniae* genomes that have been sequenced suggests they had diversified mainly by HGT in amongst the strains that were present within the studied infection. The most parsimonious explanation for the nucleotide patterns within the recombinant regions suggests that ST2011v4 acted as an extensive DNA donor during both the genesis of ST13v6 from ST13v1 and the subsequent generation of ST13v12 from ST13v6 (or ST13v6-like strains).

We were however, unable to exclude the possibility that a different, albeit less parsimonious, pattern of HGT might have yielded the observed genetic variation. Regardless of the actual recombination pathways, it was also very clear that not all of the observed exchanges could be explained by the genetic exchanges between the four sampled strains. In at least two recombination events detectable within the ST13v12 genomes sampled on visits 12 and 13 it is apparent that divergent sequences have been derived from an unsampled donor-parental lineage. Experiments with commensal populations of streptococci in the upper respiratory tract show that a few clones tend to dominate, thus it is not far fetched to suggest that there may have been multiple uncultured and undetected *S. pneumoniae* strains present within this patient during the study period [42].

The synteny in and around the recombination breakpoints, as well as the absence of phage-related sequences largely rules out transduction and suggests that HR is the most likely operative mechanism. Our detection methods are not able to differentiate between DNA acquired by conjugation or transformation. However, given that the *S. pneumoniae* are naturally competent, it is most likely that DNA from lysed bacteria enters the cells during bouts of competence, and is incorporated into the genome via homologous recombination. It is possible that each one of these 23 loci that differentiate the ST13 strains resulted from a separate HGT event and that the differences accumulated one at a time over the entire study period. Alternatively, given that at least six loci (NGs 1,3,4,7,8, and 11) were most likely all acquired by ST13v12 from ST2011v4 it is also conceivable that these regions may have been exchanged during the same competence event that supported multiple homologous recombinations. The same simultaneous multiple replacement mechanism could also be used to explain the formation of ST13v6, which differs by at least three loci (NGs 2, 5, and 12) from its most likely predecessor, ST13v1. The possibility of a single competence event resulting in the replacement of multiple loci warrants further investigation as it has not been previously explored given that *S. pneumoniae* recombination studies have been limited to a subset of genes in population-wide studies [8], [33]. If in fact, multiple loci were simultaneously replaced, the selection for one transferred gene or allelic difference would be enough to explain inheritance of the multiple replacements after a competence event. Ultimately, one would expect that the persistence over multiple generations of each of the HGT-acquired loci would depend on its adaptive value.

Our whole-genome comparisons indicate that at least 156Kb of *S. pneumoniae* strain's genomic content was exchanged during multiple HGT events involving multiple potential donors over a seven-month period. Given that the average ST13 genome is ∼2 megabases, this corresponds to ∼7.8% of the genome being replaced. Experiments in biofilm-grown *S. mutans* cells show they were transformed 10- to 600-times more frequently than their planktonic counterparts [43], suggesting that, for any given polyclonal population of bacteria, rates of recombination could be much higher in the context of a chronic biofilm infection than they would be in an analogous acute infection. The extent of homologous recombination in the *S. pneumoniae* population, estimated using theoretical models informed by MLST data (involving 6 or 7 housekeeping genes) suggests that recombination can generate new alleles ∼3–10 times more frequently than DNA polymerase errors [8], [9]. Another *S. pneumoniae* study that investigated recombination breakpoints on the seven MLST housekeeping genes led to the suggestion that some strains may have very high recombination rates -i.e. they are so-called hyper-recombinants [44]. Our whole genome data clearly support the notion that *S. pneumoniae* evolution is characterized by extremely high rates of recombination.

The unprecedented degree of HGT detected here within strains isolated from a single infection is strongly supportive of the distributed genome hypothesis [3], [27], [45]. As is the case with highly recombinogenic viral pathogens [46], the genetic malleability we have detected in *S. pneumoniae* genomes is possibly a property that this and other related bacterial species have evolved to cope with both the adaptive immunity of individual hosts and the genetic variation that exists within host populations [27], [47]. As is the case with the continuing debate over the evolutionary value of sexuality [48], [49] it may ultimately prove quite difficult to precisely enumerate costs and benefits of *S. pneumoniae*'s high rate of recombination.

## Materials and Methods

### Strains and DNA sequencing, assembly, and gene prediction

We obtained six clinical *S. pneumoniae* isolates from a pediatric patient participating in a vaccine trial at the Children's Hospital of Pittsburgh. The genomes of these strains were sequenced at the Center for Genomic Sciences (CGS) using a 454 Life Sciences FLX sequencer. The limitation of this sequencing method is that it may overlook a frame shift mutation when it is present within a homo-nucleotide stretch. As previously described, strains were assembled by the 454 Newbler *de novo* assembler and prediction of putative coding sequences and gene annotations were done by NCBI using the Microbial Genome Annotation Tools and Genome Annotation Pipeline [4]. The final assemblies have been deposited in GenBank, the accession numbers are: ABWQ for ST13v1, ADHN for ST2011v4, ABWB for ST13v6, ABWA for ST13v10, ABWU for ST13v12, and ABWC for ST13v13. The annotations prefixes are as follows: ST13v1-CGSSp14BS292, ST2011v4-CGSSpBS455; ST13v6-CGSSpBS457; ST13v10-CGSSpBS458, ST13v12-CGSSpBS293; and ST13v13-CGSSpBS397.

### Whole genome alignments

The multiple contigs from the final assembly of each genome were concatenated into a single fasta file using a combination of the Mauve Contig Mover utility of MAUVE 2.3 and manual rearrangements. The single fasta sequence of all 4 genomes was aligned using the progressive Mauve function from the MAUVE 2.3 package available at: http://asap.ahabs.wisc.edu/ [30]. A mapping of the contigs from the final assembly available in GenBank onto the whole genome alignments is available in Supplementary Table S4.

### Phylogenetic tree

A plylogenetic tree for the four genotypes from the same patient was constructed using the WGS alignment generated by Mauve, by maximum likelihood (using Phyml) [31] with optimal model and parameter selection carried out in RDP3 (revision 42–2; available from http://darwin.uvigo.es/rdp/rdp.html) [32].

### Gene clustering algorithm

A complete description of the algorithms used to create the orthologous clusters is given by Hogg *et al* [3].

### Single nucleotide polymorphism and insertion/deletion predictions

SNPs from the whole genome sequence were identified using the tab-delimited SNP file produced by MAUVE 2.3.

### Detection and characterization of recombination events

A specially modified version of RDP3 (revision 42–2; available from http://darwin.uvigo.es/rdp/rdp.html) capable of analyzing full-length bacterial genomes was used to identify signals of recombination and characterize specific detectable recombination events. An initial exploratory screen with 2 independent recombination signal detection methods in primary exploratory mode (RDP and MAXCHI) [50]–[51]; was followed up with a confirmatory screen with five additional methods (GENECONV, CHIMAERA, SISCAN, RECSCAN and 3SEQ [32], [51]–[54]. Other than RECSCAN and SISCAN window size settings being adjusted from their default settings to 10000 nucleotides, RDP window size settings being adjusted to 30 nucleotides, and sequences being analyzed as though linear, default settings were used throughout. Only recombination signals identified by five or more out of seven different recombination detection methods were accepted as evidence of recombination. In all cases the most probable position of recombination breakpoints was inferred with the MAXCHI method (which is the most accurate breakpoint detection method amongst the seven non-parametric methods implemented in RDP3). Phylogenetic trees were constructed from aligned regions bounded by identified recombination breakpoints. These were compared in RDP3 with phylogenetic trees constructed with the full genome alignment. Recombinant sequences were identified manually as the sequence that showed greatest positional shift with respect to the other sequences analyzed.

The RDP3 inferred recombination breakpoints are at the center of the two most terminal SNPs at each of the 5′ and 3′ edges of identified recombinant regions. The NG-inferred recombination breakpoints are at the most terminal SNPs at each of the 5′ and 3′ edges. As a consequence of this, the NG analysis yields a more conservative estimate of the size of recombinant regions then RDP3 by requiring that: 1) the vast majority of the SNPs in the recombination fragment have the same distribution pattern across the ST13 strains, and 2) the recombination edges exclusively contain a high concentration of SNPs (in the majority of cases the last three SNPs fitted into a 500 bp region, Table S1).

### Graphs of strain genic and allelic differences

22 *S. pneumoniae* strains were compared using genic and allelic difference-based graphs. Genic distances between genomes were calculated as the total commonality between strain subsets of distributed genes divided by the total number of distributed genes. Commonality included the case where both genomes either contained the distributed gene, or did not contain a given distributed gene. The commonality number was then subtracted by one to give the distance metric between two genomes [34]. Allelic distance measures between genomes are directly proportional to the percent identity among all the 1405 core alleles. The distance metrics were used to create a neighbor joining tree using the PHYLIP package (Version 3.69) [55]. The Fig tree package (Version 1.3.1) was used to visualize the tree using a midpoint root (freely available from http://tree.bio.ed.ac.uk/software/figtree/).

### MLST typing

The full sequence of the seven house-keeping genes used for MLST typing were obtained from the whole genome sequences of the six sequenced strains and by Ibis T-5000 universal biosensor technology for the two unsequenced strains [56]. The internal fragments required for typing were trimmed as directed by the *S. pneumoniae* MLST site, and were submitted to this site to determine their ST type (http://spneumoniae.mlst.net/).

### Serotyping

The strain serotypes were determined by two methods: (1) the Pneumotest-Latex kit (Statens Serum Intitut), and (2) a PCR-based approach [57].

### Biofilm growth

*S. pneumoniae* biofilms were grown and visualized as previously described [26].

### Ethics statement

The study was approved by the Children's Hospital of Pittsburgh Human Rights Committee. They recruited healthy children aged 6 to 24 months from the hospital's primary care center and from the community at large. Research personnel informed parents in the primary care center about the study, and advertisements were placed on the radio and in the regional newspaper. Written informed consent was obtained from the parent(s) of each enrolled child. They excluded children who had been born prematurely or had a craniofacial abnormality; or who had or were living with persons who had any medical condition placing them at high risk of complications of influenza; or who had a neurologic disorder, a history of tympanostomy tube insertion, hypersensitivity to egg protein or thimerosal, or a febrile illness or severe respiratory illness within the preceding 48 hours [28].

## Supporting Information

Figure S1 Example of DNA inversion within ST13 strains. (A) Mauve generated alignment where solid colored blocks represent regions that are almost identical between ST13v1 and ST13v12. The black square surrounds the region where an inversion has occurred between the strains, leading to a “switch” in the C-terminal ends of restriction endonuclease S subunits located near each other on the chromosome. (B) Alignment of 4 genes, two from strain ST13v1 (prefix:CGSSp14BS292) and two from ST13v12 (prefix: CGSSpBS293). Yellow, blue, red, pink, and gray highlight identical regions. Comparison between both strains suggests that sequence differences were created by site-specific DNA inversion systems, where DNA inversions occurred within the coding regions of restriction endonuclease subunits resulting in genetic polymorphism, as previously observed in *S. pneumoniae* [35].
(0.48 MB PPT)

Table S1 List of SNPs that differentiate strains isolated from patient 19. The first 3306 rows are loci that contain at least one SNP among theST13 strains; the remaining rows are loci that are identical among the ST13 strains, but are variable relative to ST2011v4. QS =  sequencing quality score.
(3.98 MB XLS)

Table S2 List and annotation of 173 distributed clusters that differentiate the strains isolated from patient 19. 47 of these vary among the ST13 strains (purple, pink, blue and yellow).
(0.07 MB XLS)

Table S3 List of the coding sequences within the NGs (W) as well as those in the surrounding regions (S) that contain allelic differences among ST13 strains. c = complement.
(0.92 MB XLS)

Table S4 Map of the contigs from ST13v1, ST13v6, ST13v12, and ST2011v4 onto the assembly used for whole genome alignments.
(0.04 MB XLS)

Text S1 Comparison of the strains ST13v12 and ST13v13 reveals no evidence of HGT.
(3.49 MB DOC)

Text S2 Comparison of the strains ST13v1 and ST13v10 reveals no evidence of HGT.
(5.22 MB DOC)

Text S3 Main differences between the RDP3 and NG predictions.
(0.02 MB DOC)

## References

[1] Gogarten JP, Townsend JP (2005). Horizontal gene transfer, genome innovation and evolution.. Nat Rev Microbiol.

[2] Tettelin H, Masignani V, Cieslewicz MJ, Donati C, Medini D (2005). Genome analysis of multiple pathogenic isolates of Streptococcus agalactiae: implications for the microbial “pan-genome”.. Proc Natl Acad Sci U S A.

[3] Hogg JS, Hu FZ, Janto B, Boissy R, Hayes J (2007). Characterization and modeling of the Haemophilus influenzae core and supragenomes based on the complete genomic sequences of Rd and 12 clinical nontypeable strains.. Genome Biol.

[4] Hiller NL, Janto B, Hogg JS, Boissy R, Yu S (2007). Comparative Genomic Analyses of Seventeen Streptococcus pneumoniae Strains: Insights into the Pneumococcal Supragenome.. J Bacteriol.

[5] Smith JM, Dowson CG, Spratt BG (1991). Localized sex in bacteria.. Nature.

[6] Retchless AC, Lawrence JG (2007). Temporal fragmentation of speciation in bacteria.. Science.

[7] Smith NH, Dale J, Inwald J, Palmer S, Hewinson RG (2003). The population structure of Mycobacterium bovis in Great Britain: clonal expansion.. Proc Natl Acad Sci U S A.

[8] Feil EJ, Smith JM, Enright MC, Spratt BG (2000). Estimating recombinational parameters in Streptococcus pneumoniae from multilocus sequence typing data.. Genetics.

[9] Fraser C, Hanage WP, Spratt BG (2005). Neutral microepidemic evolution of bacterial pathogens.. Proc Natl Acad Sci U S A.

[10] Brochet M, Rusniok C, Couvé E, Dramsi S, Poyart C (2008). Shaping a bacterial genome by large chromosomal replacements, the evolutionary history of Streptococcus agalactiae.. Proc Natl Acad Sci U S A.

[11] Schwarz S, Morelli G, Kusecek B, Manica A, Balloux F (2008). Horizontal versus familial transmission of Helicobacter pylori.. PLoS Pathog.

[12] Ehrlich GD, Ahmed A, Earl J, Hiller NL, Costerton JW (2010). The Distributed Genome Hypothesis as a Rubric for Understanding Evolution in situ During Chronic Bacterial Biofilm Infectious Processes.. FEMS Immunol Med Microbiol.

[13] Post JC, Preston RA, Aul JJ, Larkins-Pettigrew M, Rydquist-White J (1995). Molecular analysis of bacterial pathogens in otitis media with effusion.. JAMA.

[14] WHO, W.H.O. (2008). http://www.who.int/vaccine_research/diseases/ari/en/index5.html.

[15] Sa-Leao R, Nunes S, Brito-Avô A, Alves CR, Carriço JA (2008). High rates of transmission of and colonization by Streptococcus pneumoniae and Haemophilus influenzae within a day care center revealed in a longitudinal study.. J Clin Microbiol.

[16] St Sauver J, Marrs CF, Foxman B, Somsel P, Madera R (2000). Risk factors for otitis media and carriage of multiple strains of Haemophilus influenzae and Streptococcus pneumoniae.. Emerg Infect Dis.

[17] Bentley SD, Aanensen DM, Mavroidi A, Saunders D, Rabbinowitsch E (2006). Genetic analysis of the capsular biosynthetic locus from all 90 pneumococcal serotypes.. PLoS Genet.

[18] Park IH, Pritchard DG, Cartee R, Brandao A, Brandileone MC (2007). Discovery of a new capsular serotype (6C) within serogroup 6 of Streptococcus pneumoniae.. J Clin Microbiol.

[19] Forbes ML, Horsey E, Hiller NL, Buchinsky FJ, Hayes JD (2008). Strain-specific virulence phenotypes of Streptococcus pneumoniae assessed using the Chinchilla laniger model of otitis media.. PLoS ONE.

[20] Shen K, Wang X, Post JC, Ehrlich GD, Rosenfield R, Bluestone CD (2003). Molecular and Translational Research Approaches for the Study of Bacterial Pathogenesis in Otitis Media..

[21] Shen K, Gladitz J, Antalis P, Dice B, Janto B (2006). Characterization, distribution, and expression of novel genes among eight clinical isolates of Streptococcus pneumoniae.. Infect Immun.

[22] Whitchurch CB, Tolker-Nielsen T, Ragas PC, Mattick JS (2002). Extracellular DNA required for bacterial biofilm formation.. Science.

[23] Dawid S, Roche AM, Weiser JN (2007). The blp bacteriocins of Streptococcus pneumoniae mediate intraspecies competition both in vitro and in vivo.. Infect Immun.

[24] Claverys JP, Prudhomme M, Martin B (2006). Induction of competence regulons as a general response to stress in gram-positive bacteria.. Annu Rev Microbiol.

[25] Håvarstein LS, Martin B, Johnsborg O, Granadel C, Claverys JP (2006). New insights into the pneumococcal fratricide: relationship to clumping and identification of a novel immunity factor.. Mol Microbiol.

[26] Hall-Stoodley L, Nistico L, Sambamthamoorthy K, Dice B, Nguyen D (2008). Characterization of biofilm matrix, degradation by DNase treatment and evidence of capsule downregulation in Streptococcus pneumoniae clinical isolates.. BMC Microbiol.

[27] Hu FZ, Ehrlich GD (2008). Population-level virulence factors amongst pathogenic bacteria: relation to infection outcome.. Future Microbiol.

[28] Hoberman A, Greenberg DP, Paradise JL, Rockette HE, Lave JR (2003). Effectiveness of inactivated influenza vaccine in preventing acute otitis media in young children: a randomized controlled trial.. JAMA.

[29] Dagerhamn J, Blomberg C, Browall S, Sjöström K, Morfeldt E (2008). Determination of accessory gene patterns predicts the same relatedness among strains of Streptococcus pneumoniae as sequencing of housekeeping genes does and represents a novel approach in molecular epidemiology.. J Clin Microbiol.

[30] Darling AE, Treangen TJ, Messeguer X, Perna NT (2007). Analyzing patterns of microbial evolution using the mauve genome alignment system.. Methods Mol Biol.

[31] Guindon S, Gascuel O (2003). A simple, fast, and accurate algorithm to estimate large phylogenies by maximum likelihood.. Syst Biol.

[32] Martin DP, Williamson C, Posada D (2005). RDP2: recombination detection and analysis from sequence alignments.. Bioinformatics.

[33] Feil EJ, Holmes EC, Bessen DE, Chan MS, Day NP (2001). Recombination within natural populations of pathogenic bacteria: short-term empirical estimates and long-term phylogenetic consequences.. Proc Natl Acad Sci U S A.

[34] Hall BG, Ehrlich GD, Hu FZ (2010). Pan-genome analysis provides much higher strain typing resolution than does MLST.. Microbiology.

[35] Dybvig K, Sitaraman R, French CT (1998). A family of phase-variable restriction enzymes with differing specificities generated by high-frequency gene rearrangements.. Proc Natl Acad Sci U S A.

[36] Hall-Stoodley L, Hu FZ, Gieseke A, Nistico L, Nguyen D (2006). Direct detection of bacterial biofilms on the middle-ear mucosa of children with chronic otitis media.. JAMA.

[37] Ehrlich GD, Veeh R, Wang X, Costerton JW, Hayes JD (2002). Mucosal biofilm formation on middle-ear mucosa in the chinchilla model of otitis media.. JAMA.

[38] Tu AH, Fulgham RL, McCrory MA, Briles DE, Szalai AJ (1999). Pneumococcal surface protein A inhibits complement activation by Streptococcus pneumoniae.. Infect Immun.

[39] Hammerschmidt S, Bethe G, Remane PH, Chhatwal GS (1999). Identification of pneumococcal surface protein A as a lactoferrin-binding protein of Streptococcus pneumoniae.. Infect Immun.

[40] Griffith F (1928). The significance of Pneumococcal Types.. The Journal of Hygiene.

[41] Avery OT, MacLeod CM, McCarty M (1944). Studies on the chemical nature of the substance inducing transformation of pneumococcal types. Inductions of transformation by a desoxyribonucleic acid fraction isolated from pneumococcus type III.. J Exp Med.

[42] Bek-Thomsen M, Tettelin H, Hance I, Nelson KE, Kilian M (2008). Population diversity and dynamics of Streptococcus mitis, Streptococcus oralis, and Streptococcus infantis in the upper respiratory tracts of adults, determined by a nonculture strategy.. Infect Immun.

[43] Li YH, Lau PC, Lee JH, Ellen RP, Cvitkovitch DG (2001). Natural genetic transformation of Streptococcus mutans growing in biofilms.. J Bacteriol.

[44] Hanage WP, Fraser C, Tang J, Connor TR, Corander J (2009). Hyper-recombination, diversity, and antibiotic resistance in pneumococcus.. Science.

[45] Ehrlich GD, Hu FZ, Shen K, Stoodley P, Post JC (2005). Bacterial plurality as a general mechanism driving persistence in chronic infections.. Clin Orthop Relat Res.

[46] Onafuwa-Nuga A, Telesnitsky A (2009). The remarkable frequency of human immunodeficiency virus type 1 genetic recombination.. Microbiol Mol Biol Rev.

[47] Ehrlich GD, Hiller NL, Hu FZ (2008). What makes pathogens pathogenic.. Genome Biol.

[48] Maynard Smith J (1978). The Evolution of Sex..

[49] Kondrashov AS (1988). Deleterious mutations and the evolution of sexual reproduction.. Nature.

[50] Martin D, Rybicki E (2000). RDP: detection of recombination amongst aligned sequences.. Bioinformatics.

[51] Smith JM (1992). Analyzing the mosaic structure of genes.. J Mol Evol.

[52] Posada D, Crandall KA (2001). Evaluation of methods for detecting recombination from DNA sequences: computer simulations.. Proc Natl Acad Sci U S A.

[53] Gibbs MJ, Armstrong JS, Gibbs AJ (2000). Sister-scanning: a Monte Carlo procedure for assessing signals in recombinant sequences.. Bioinformatics.

[54] Boni MF, Posada D, Feldman MW (2007). An exact nonparametric method for inferring mosaic structure in sequence triplets.. Genetics.

[55] Felsenstein J (1989). PHYLIP - Phylogeny Inference Package (Version 3.2).. Cladistics.

[56] Ecker DJ, Sampath R, Massire C, Blyn LB, Hall TA (2008). Ibis T5000: a universal biosensor approach for microbiology.. Nat Rev Microbiol.

[57] Pai R, Gertz RE, Beall B (2006). Sequential multiplex PCR approach for determining capsular serotypes of Streptococcus pneumoniae isolates.. J Clin Microbiol.

