# Supplementary material for: Generation of Genic Diversity among Streptococcus pneumoniae Strains via Horizontal Gene Transfer during a Chronic Polyclonal Pediatric Infection
Source: PLoS Pathog. 2010 Sep 16;6(9):e1001108. doi: 10.1371/journal.ppat.1001108 (PMC2940740; doi:10.1371/journal.ppat.1001108)
Supplement: Figure S1 — Example of DNA inversion within ST13 strains. (A) Mauve generated alignment where solid colored blocks represent regions that are almost identical between ST13v1 and ST13v12. The black square surrounds the region where an inversion has occurred between the strains, leading to a “switch” in the C-terminal ends of restriction endonuclease S subunits located near each other on the chromosome. (B) Alignment of 4 genes, two from strain ST13v1 (prefix:CGSSp14BS292) and two from ST13v12 (prefix: CGSSpBS293). Yellow, blue, red, pink, and gray highlight identical regions. Comparison between both strains suggests that sequence differences were created by site-specific DNA inversion systems, where DNA inversions occurred within the coding regions of restriction endonuclease subunits resulting in genetic polymorphism, as previously observed in S. pneumoniae [35]. (0.48 MB PPT) [file ppat.1001108.s001.ppt]

## Slide 1
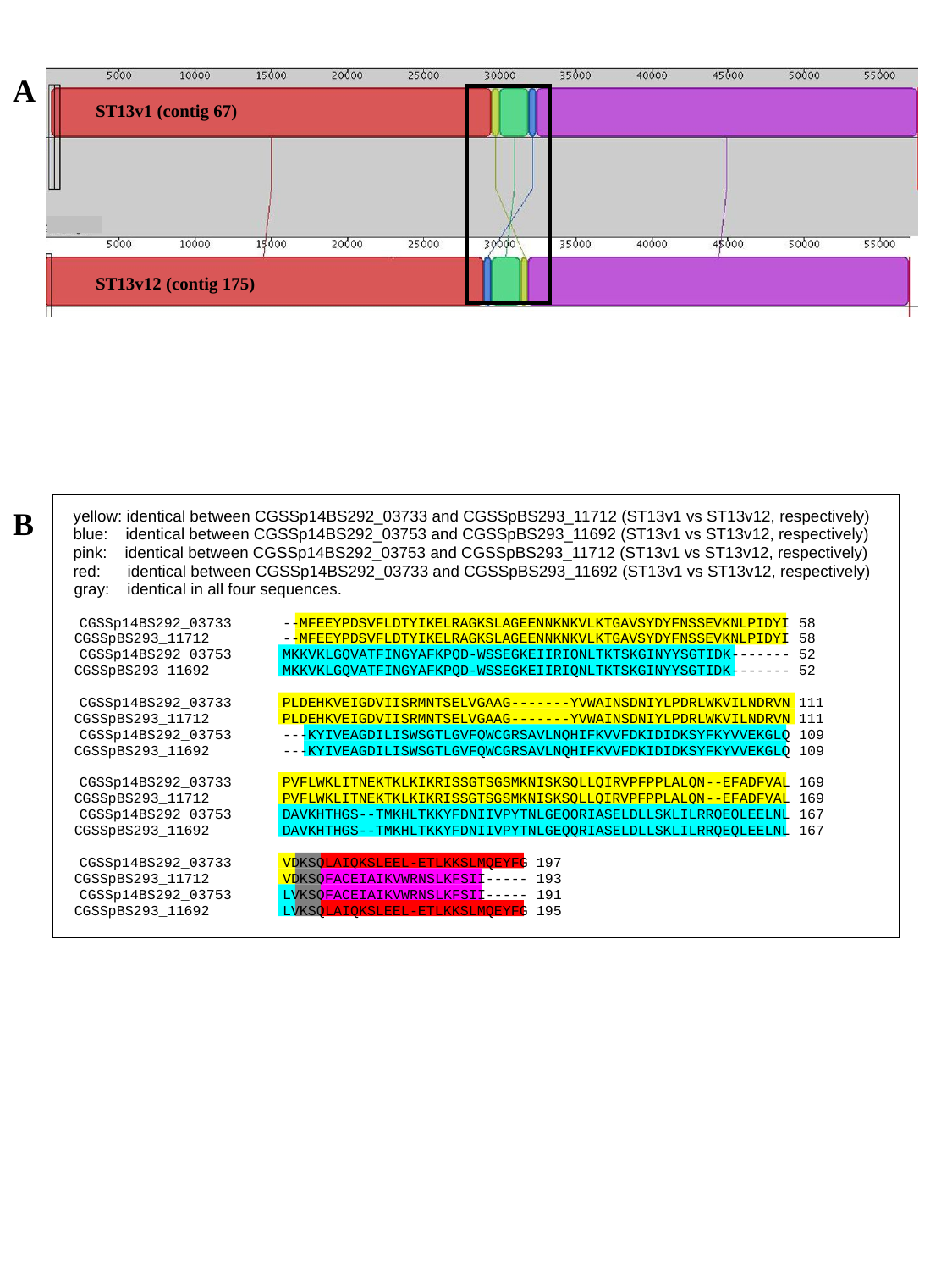

A
ST13v1 (contig 67)
ST13v12 (contig 175)
B
yellow: identical between CGSSp14BS292_03733 and CGSSpBS293_11712 (ST13v1 vs ST13v12, respectively)
blue: identical between CGSSp14BS292_03753 and CGSSpBS293_11692 (ST13v1 vs ST13v12, respectively)
pink: identical between CGSSp14BS292_03753 and CGSSpBS293_11712 (ST13v1 vs ST13v12, respectively)
red: identical between CGSSp14BS292_03733 and CGSSpBS293_11692 (ST13v1 vs ST13v12, respectively)
gray: identical in all four sequences.
CGSSp14BS292_03733
--
MFEEYPDSVFLDTYIKELRAGKSLAGEENNKNKVLKTGAVSYDYFNSSEVKNLPIDYI
 58
CGSSpBS293_11712
--
MFEEYPDSVFLDTYIKELRAGKSLAGEENNKNKVLKTGAVSYDYFNSSEVKNLPIDYI
 58
CGSSp14BS292_03753
MKKVKLGQVATFINGYAFKPQD
-
WSSEGKEIIRIQNLTKTSKGINYYSGTID
K
-
------
 52
CGSSpBS293_11692
MKKVKLGQVATFINGYAFKPQD
-
WSSEGKEIIRIQNLTKTSKGINYYSGTIDK
-
------
 52
CGSSp14BS292_03733
PLDEHKVEIGDVIISRMNTSELVGAAG
-------
YVWAINSDNIYLPDRLWKVILNDRVN
111
CGSSpBS293_11712
PLDEHKVEIGDVIISRMNTSELVGAAG
-------
YVWAINSD
NIYLPDRLWKVILNDRVN
111
CGSSp14BS292_03753
---
KYIVEAGDILISWSGTLGVFQWCGRSAVLNQHIFKVVFDKIDIDKSYFKYVVEKGLQ
 109
CGSSpBS293_11692
---
KYIVEAGDILISWSGTLGVFQWCGRSAVLNQHIFKVVFDKIDIDKSYFKYVVEKGLQ
 109
CGSSp14BS292_03733
PVFLWKLITNEKTKLKIKRISSGTSGSMKN
ISKSQLLQIRVPFPPLALQN
--
EFADFVAL
 169
CGSSpBS293_11712
PVFLWKLITNEKTKLKIKRISSGTSGSMKNISKSQLLQIRVPFPPLALQN
--
EFADFVAL
 169
CGSSp14BS292_03753
DAVKHTHGS
--
TMKHLTKKYFDNIIVPYTNLGEQQRIASELDLLSKLILRRQEQLEELNL
 167
CGSSpBS293_11692
DAVKHTHGS
--
TMKHLTKK
YFDNIIVPYTNLGEQQRIASELDLLSKLILRRQEQLEELNL
 167
CGSSp14BS292_03733
VD
KSQ
LAIQKSLEEL
-
ETLKKSLMQEYFG
 197
CGSSpBS293_11712
VD
KSQ
FACEIAIKVWRNSLKFSII
-----
 193
CGSSp14BS292_03753
LV
KSQ
FACEIAIKVWRNSLKFSII
-----
 191
CGSSpBS293_11692
LV
KSQ
LAIQKS
LEEL
-
ETLKKSLMQEYFG
 195
