# Supplementary material for: Generation of Genic Diversity among Streptococcus pneumoniae Strains via Horizontal Gene Transfer during a Chronic Polyclonal Pediatric Infection
Source: PLoS Pathog. 2010 Sep 16;6(9):e1001108. doi: 10.1371/journal.ppat.1001108 (PMC2940740; doi:10.1371/journal.ppat.1001108)
Supplement: Text S1 — Comparison of the strains ST13v12 and ST13v13 reveals no evidence of HGT. (3.49 MB DOC) [file ppat.1001108.s006.doc]

**Text S1:** Comparison of the strains isolated at the last two visits, ST13v12 and ST13v13 reveals no evidence of HGT.

Strains ST13v13 and ST13v12 have the same set of orthologous clusters. Global alignment of their entire genomes using NUCmer (MUMMER package) was performed to identify any mismatched regions between the multiple contigs from each of these genomes. This analysis shows only minor difference between these two strains. Importantly, all of the other mismatched regions (the sum of all the gaps/insertions is 2093bp) occur at the start or end of one of the contigs being aligned, consistent with the probability that these are areas where genome closure is incomplete and not areas with true differences amongst the strains (A).

To further inspect differences between these genomes, all contigs from both genomes were aligned in Sequencher and visually inspected for mismatched regions. Four regions with possible differences in lengths were identified by this approach, but polymerase chain reaction (PCR) based Sanger sequencing verified only a single region that encodes the pneumococcal surface protein A (PspA) gene as being different. This gene contains various numbers of an 8-residue repeat with ST13v12 containing six and ST13v13 twelve (B).

Finally, to investigate whether the genically identical strains ST13v12 and ST13v13 have allelic differences we used NUCmer to compare the CDSs between these strains. We identified only 30 CDSs with SNPs, and no groups of 3 or more CDSs that cluster together (C). The small number of dispersed SNPs suggests that multi-gene regions have not been exchanged between these strains and thus this unencapsulated strain has persisted with only replicative errors in this patient over a 3-week period.

A. Global alignment of ST13v12 and ST13v13 genomes using NUCmer.

B. Difference in PspA gene between ST13v12 and ST13v13.

Blue: primers used for PCR based Sanger sequencing. Pink: region present in ST13v13 (contig 110) and missing in ST13v12 (contig 90). This region consists of 216 bp that belong to the repeat region of PspA.

AGTTCCAGGCGCTTTATGAGTCAACTCAAGAACAAATCGAAGAGTTGAAA**GACTACAACGAACAAATCTCTGAAGGA**GAAGAAGCCCTTATCTCTGCTCTTCAAAATAAAATCTCAGACCTTGATGATAAAATCGCAGAAGCGGAGAAAAATCTTGCTGACTCACAAAATGGAGAGGCTGTAGAAGACTATTATACTTCAGGTGATAAGGACAAATTAGAAAAGCTTCAAGCAGAGCAAGATGAACTTCAAGCTGAATTGGATCAATTGTTGGATGAAGTTGATGGACAAGAACCAGCTCCTGCACCACAACCAGAGCAACCAGCTCCTGCACCACAACCAGAGCAACCAGCTCCTGCACCACAACCAGAGCAACCAGCTCCTGCACCACAACCAGAGCAACCAGCTCCTGCACCACAACCAGAGCAACCAGCTCCTGCACCACAACCAGAGCAACCAGCTCCTGCACCA**CAACCAGAGCAACCAGCTCCTGCACCACAACCAGAGCAACCAGCTCCTGCACCACAACCAGAGCAACCAGCTCCTGCACCACAACCAGAGCAACCAGCTCCAGCACCACAACCAGAGCAACCAGCTCCTGCACCACAACCAGAGCAACCAGCTCCTGCACCACAACCAGAGCAACCAGCTCCTGCACCACAACCAGAGCAACCAGCTCCTGCACCA**AAAATAGGCTGGAAACAAGAAAACGGTATGTGGTACTTCTACAATACT**GATGGTTCAATGGCGACAGGTT**GGCTACAAAACAACGGTTCATGGTACTACCTAAACGCTAACGGCGCTATG

C. Comparison of CDSs between ST13v12 and ST13v13 using NUCmer to identify single nucleotide polymorphisms and frame shifts
