# Supplementary material for: Generation of Genic Diversity among Streptococcus pneumoniae Strains via Horizontal Gene Transfer during a Chronic Polyclonal Pediatric Infection
Source: PLoS Pathog. 2010 Sep 16;6(9):e1001108. doi: 10.1371/journal.ppat.1001108 (PMC2940740; doi:10.1371/journal.ppat.1001108)
Supplement: Text S2 — Comparison of the strains ST13v1 and ST13v10 reveals no evidence of HGT. (5.22 MB DOC) [file ppat.1001108.s007.doc]

**Text S2:** Comparison of the strains ST13v1 and ST13v10 reveals no evidence of HGT.

There are no genic differences between strains ST13v1 and ST13v10. Note that *in silico* genic comparisons between these two strains by orthologous clustering revealed two CDSs missing in ST13v10, however PCR-based sequence analysis showed that these CDSs are in assembly gaps and thus these strains are most likely genically identical (A). To establish whether there are allelic differences between these strains, we used NUCmer to compare their CDSs. This analysis identified 13 SNPs and no groups of 3 or more CDSs grouped on the contigs (B). A global alignment between these strains using NUCmer shows that 160 bp distributed over 3 regions are present in ST13v10 and missing in ST13v1, and ~13 Kb distributed over 64 regions are present in ST13v1 and missing in ST13v10. The median size of the ST13v10 gaps is 136bp, and the longest gap is 1321bp (C). It is possible that these gaps correspond to small insertions in the ST13v1 genome relative to ST13v10, however since the genome of ST13v10 is distributed over 109 contigs and the mismatched regions are scattered over many small regions, it's is most likely that these correspond to gaps in the ST13v10 genome assembly. These genic, allelic, and global comparisons of the genomes of isolates ST13v1 and ST13v10 shows that they represent a single strain that has persisted in this patient for 5 months without any evidence of HGT.

A. Primers used in PCR reaction to establish that there are no genic differences between ST13v1 and ST13v10.

B. Comparison of CDSs between ST13v1 and ST13v10 to identify single nucleotide polymorphisms and frame shifts using NUCmer.

C. Whole Genome alignment between ST13v1 and ST13v10 using NUCmer.
