# Supplementary material for: Generation of Genic Diversity among Streptococcus pneumoniae Strains via Horizontal Gene Transfer during a Chronic Polyclonal Pediatric Infection
Source: PLoS Pathog. 2010 Sep 16;6(9):e1001108. doi: 10.1371/journal.ppat.1001108 (PMC2940740; doi:10.1371/journal.ppat.1001108)
Supplement: Text S3 — Main differences between the RDP3 and NG predictions. (0.02 MB DOC) [file ppat.1001108.s008.doc]

**Text S3**: Main differences between the RDP3 and NG predictions.

The prominent differences between the predictions by RDP3 and NG methods are confined to the position of the recombination breakpoints in three regions. Two of these where identified by RDP as a single recombination block, but NG analysis suggested two distinct events. In the remaining case RDP split a single NG block into three recombination regions (Table 5).

In the first region, NG1 (0.7Kb) and NG2 (4Kb) are within the much larger RDP3-predicted 36Kb region A. In the second region, NG9 (0.9Kb) and the 3' section of adjacent NG8 (19Kb) are combined and extended into the 235 Kb RDP3-predicted region H. Finally, NG curation treats NG14 as one 56 Kb event, while RDP splits it into the 3 tandem regions N (4.7Kb), O (2 Kb), and P (47.9Kb).
